# Schwannome malin historique de l'avant bras

**DOI:** 10.11604/pamj.2015.21.98.6894

**Published:** 2015-06-09

**Authors:** Ismail Hmouri, Mohamed Kharmaz

**Affiliations:** 1 Clinique de Chirurgie Orthopédique, CHU Avicenne, Rabat, Maroc

**Keywords:** Tumeur, nerf, maligne, Tumor, nerve, malignant

## Image en medicine

Il s'agit d'un jeune homme de 33 ans, sans antécédents pathologiques notables, qui consulte pour une énorme tumeur (A) bourgeonnante et ulcérée de l'avant bras droit évoluant depuis 3 ans. Une biopsie a été faite ayant conclue pour une tumeur nerveuse de haut grade de malignité. L'IRM (B) a montré une tumeur mesurant 141/64/69mm correspondant à un neurofibrosarcome du nerf interosseux postérieur. La prise en charge thérapeutique du patient a consisté en une amputation au niveau du tiers inferieur du bras (C).

**Figure 1:**
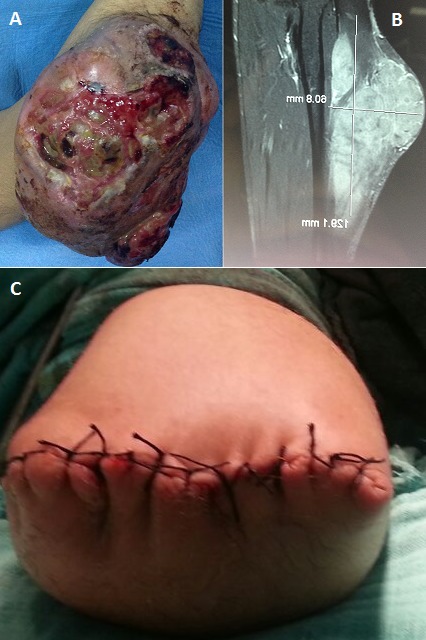
(A) image clinique montrant la tumeur ulcéro-bourgeonnante de l'avant bras; (B) image IRM montrant la tumeur; (C) moignon d'amputation

